# The taxonomy of Cyclospora.

**DOI:** 10.3201/eid0304.970426

**Published:** 1997

**Authors:** W C Marquardt


9. Basta M, Karmali M, Lingwood C. Sensitive receptor-specified enzyme-linked immunosorbent assay for *Escherichia coli* verocytotoxin. J Clin Microbiol 1989;27:1617-22.
10. Gunzer F, Bohm H, Russmann H, Bitzan M, Aleksic S, Karch H. Molecular detection of sorbitol-fermenting *Escherichia coli* O157 in patients with hemolytic-uremic syndrome. J Clin Microbiol 1992;30:1807-10.

### The Taxonomy of *Cyclospora*

**To the Editor:** In the article by N.J. Pieniazek and B.L. Herwaldt (1) on the rRNA gene of *Cyclospora cayetanensis,* the authors suggest that *Cyclospora* should be placed in the genus *Eimeria* because the rRNA genes of the two genera have similar sequences. The article refers to Norman D. Levine's chapter on the Apicomplexa in the Illustrated Guide to the Protozoa (2). Regrettably, the authors failed to read the whole chapter and to recognize that the initial characteristics for placing the oocyst of a coccidium in its proper genus are the number of sporocysts and then the number of sporozoites in each sporocyst. The genus *Eimeria* has four sporocysts and two sporozoites in each sporocyst. The genus *Cyclospora* has two sporocysts, each of which has two sporozoites.

The original taxonomists (3) of *C. cayetanensis* recognized that it should be placed in the taxonomic family Eimeriidae, close to *Eimeria*, but they adhered to the traditional designation for genera of coccidia. Pieniazek and Herwaldt should be cognizant of the rules of zoologic nomenclature as well as the fact that certain morphologic characteristics of protists have served us well for many decades and continue to be useful. There are serious consequences to changing the classification of an organism, and it should not be thought that one can make such a change casually. I encourage the editors of Emerging Infectious Diseases to seek the advice of those who understand what should be done with respect to the classification and nomenclature of organisms.

**William C. Marquardt**
Colorado State University, Fort Collins, Colorado, USA

### References

1. Pieniazek NJ, Herwaldt BL. Reevaluating the molecular taxonomy: Is human-associated *Cyclospora* a mammalian *Eimeria* species? Emerg Infect Dis 1997;3:381-3.
2. Levine ND. Apicomplexa. In: Lee JJ, Hutner SH, Boyce EC, editors. An illustrated guide to the protozoa. Lawrence (KS): Society of Protozoologists; 1985. p.322-74.
3. Ortega YR, Gilman RH, Steling CR. A new coccidian parasite (Apicomplexa:Eimeriidae) from humans. J Parasitol 1994;80:625-9.

**Reply to W.C. Marquardt:** Dr. Marquardt's advocacy for reliance on morphologic characteristics even if phylogenetic data become available that lead to a different conclusion runs counter to that expressed in an article he coauthored, which supports the importance of molecular data (1). The introduction of that paper states the following:

"Early systematists relied largely on light microscopic structures and life cycle patterns to separate protozoa taxonomically.... Apicomplexans display enormous variations in life cycle patterns, physiology, cytology, and biochemistry. There is no consensus on which characteristics should be relied upon to infer phylogenetic relationships. Developmental and ultrastructural features have been used to infer evolutionary relationships among representative genera in the class Sporozoea. However, comparisons of phenotypic characters are qualitative and lack objective quantitative assessment to infer genetic relationships. Sequence similarities between proteins or genes which share a common evolutionary history can be used to infer quantitative phylogenetic relationships. The small subunit (16S-like) rRNAs and their coding regions are especially useful for estimating the extent of genetic relatedness over broad evolutionary ranges."

That paper concludes with the statement that "ribosomal RNA sequence analyses of other apicomplexans are required in order to test the validity of relationships inferred from structures and life cycle patterns." Similarly, we concluded our paper as follows: "Reports based on morphologic features alone may suffer from poor resolution of features needed for classification of closely related

